# Improving Loop Modeling of the Antibody Complementarity-Determining Region 3 Using Knowledge-Based Restraints

**DOI:** 10.1371/journal.pone.0154811

**Published:** 2016-05-16

**Authors:** Jessica A. Finn, Julia Koehler Leman, Jordan R. Willis, Alberto Cisneros, James E. Crowe, Jens Meiler

**Affiliations:** 1 Department of Pathology, Microbiology and Immunology, Vanderbilt University, Nashville, Tennessee, United States of America; 2 Department of Chemistry, Vanderbilt University, Nashville, Tennessee, United States of America; 3 Department of Pediatrics, Vanderbilt University, Nashville, Tennessee, United States of America; 4 Department of Pharmacology, Vanderbilt University, Nashville, Tennessee, United States of America; 5 Chemical and Physical Biology Program, Vanderbilt University, Nashville, Tennessee, United States of America; 6 Vanderbilt Vaccine Center, Vanderbilt University, Nashville, Tennessee, United States of America; 7 Department of Chemical and Biomolecular Engineering, Johns Hopkins University, Baltimore, Maryland, United States of America; National Cancer Institute, NIH, UNITED STATES

## Abstract

Structural restrictions are present even in the most sequence diverse portions of antibodies, the complementary determining region (CDR) loops. Previous studies identified robust rules that define canonical structures for five of the six CDR loops, however the heavy chain CDR 3 (HCDR3) defies standard classification attempts. The HCDR3 loop can be subdivided into two domains referred to as the “torso” and the “head” domains and two major families of canonical torso structures have been identified; the more prevalent “bulged” and less frequent “non-bulged” torsos. In the present study, we found that Rosetta loop modeling of 28 benchmark bulged HCDR3 loops is improved with knowledge-based structural restraints developed from available antibody crystal structures in the PDB. These restraints restrict the sampling space Rosetta searches in the torso domain, limiting the φ and ψ angles of these residues to conformations that have been experimentally observed. The application of these restraints in Rosetta result in more native-like structure sampling and improved score-based differentiation of native-like HCDR3 models, significantly improving our ability to model antibody HCDR3 loops.

## Introduction

The field of antibody-mediated immunity has long benefited from structural studies of protein-protein interactions, in most cases through the determination of co-crystal structures of antibodies in complex with their antigens. Such studies often reveal the molecular mechanism of pathogen neutralization [1–4]. However, the size and complexity of the antibody repertoire coupled with the substantial resources needed for experimental structure determination prohibit such studies on a comprehensive scale. B cell development leads to the generation of a large population of unique antibody proteins, and it is theorized that this diverse antibody repertoire may contain 10^11^ or more different protein sequences [5,6]. Recent studies determined that the circulating antibody repertoire contains at least 10^6^ unique sequences, a number still far too large for comprehensive experimental structural studies [7,8].

Analysis of antibody structures determined by X-ray crystallography revealed conservation of structural features even in the regions of the antibody with the most sequence diversity, the six complementarity determining region (CDR) loops, which are responsible for antigen binding. Three of these loops are contributed by the heavy chain component of the fragment variable (Fv) domain of the antibody (HCDRs), and three are contributed by the light chain Fv domain (LCDRs). Two studies have identified robust rules that define canonical structures for five of the six CDR loops [9,10]. However, the HCDR3 defies classification attempts. The HCDR3 is encoded by the junction of three gene segments (V, D and J genes) connected by random nucleotide additions or deletions that are not encoded in the antibody germline gene segments, but rather introduced by the host enzyme terminal deoxynucleotidyl transferase during antibody gene recombination. The HCDR3 is therefore significantly more diverse in sequence length and composition than the other CDR loops, which are encoded by either a single gene segment (heavy and light chain CDRs 1 and 2) or by a simplified junction (LCDR3) [11–13]. As a result a large and diverse conformational space is observed for HCDR3s. Accordingly, HCDR3 is often especially important for antigen recognition and binding as has been revealed in previous structural studies [14].

The Rosetta software suite for macromolecular modeling can *de novo* predict the structure of a protein or portions thereof. The tertiary structure of a protein is determined from its primary sequence by pairing effective sampling techniques with knowledge-based energy functions. These energy functions for the most part assume that optimal geometries within proteins can be derived from a statistical analysis of the available structural information stored in the Protein Data Bank [15,16]. Similar approaches are used during comparative modeling, when structurally divergent regions (typically loops) of otherwise homologous proteins must be predicted [17]. Rosetta is capable of predicting antibody structures with low root mean square deviation (RMSD) to experimental structures outside the HCDR3; however accurately modeling the HCDR3 loop remains a challenge [18–21].

**Fig 1:**
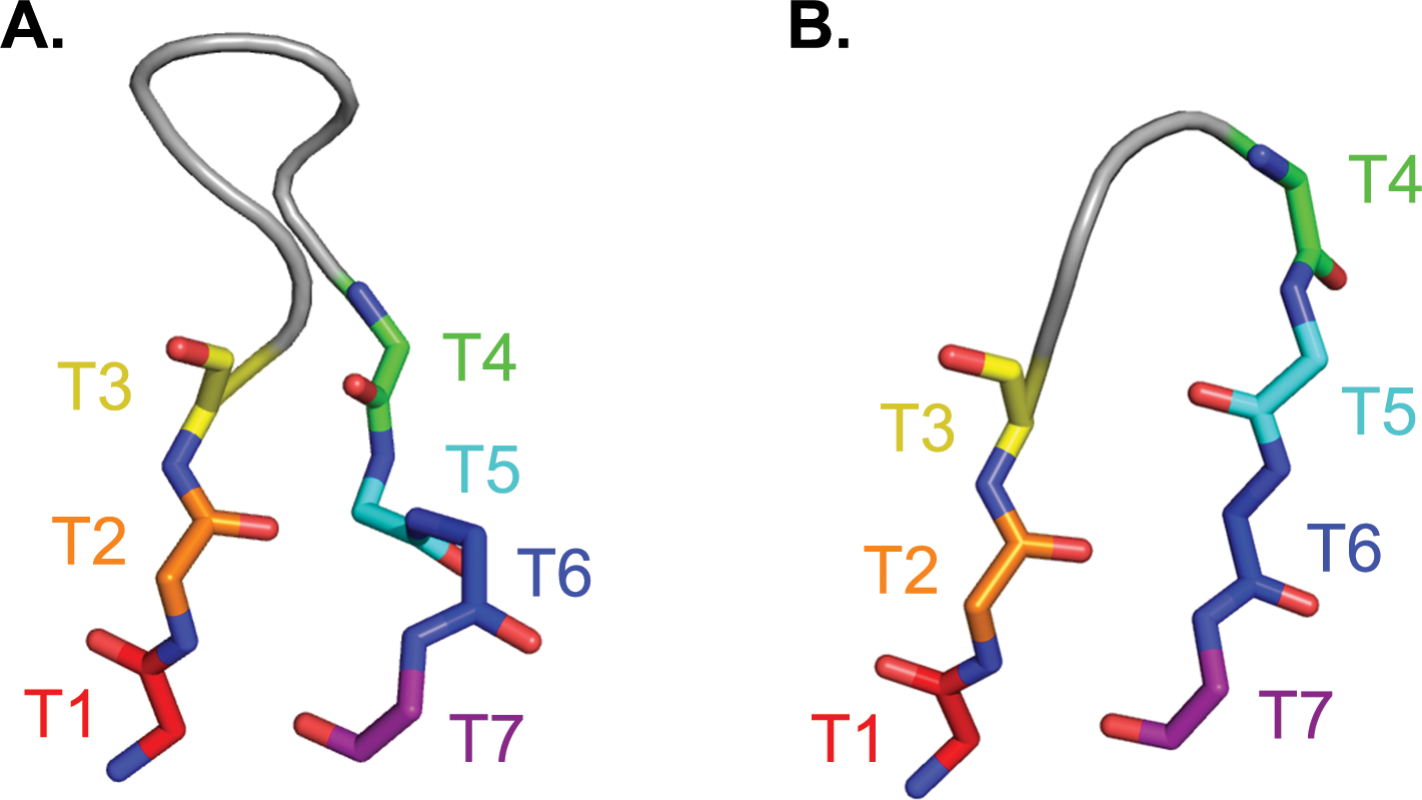
Defining the HCDR3 torso. The torso is defined as the first three and last four residues of the HCDR3 loop, numbered from T1 to T7. Main chain atoms are shown for bulged (panel A; PDBID 1UYW) and non-bulged (panel B; PDBID 2J88) torsos. In many (but not all) bulged torsos, a side-chain interaction between T2 and T6 causes the C-terminal side of the torso to bulge outward; the lack of such an interaction in non-bulged torsos leaves the beta-strand structure intact.

In an effort to classify canonical structures of the HCDR3 loop, prior work has subdivided it into two domains: the less diverse “torso” and the more variable “head” (Fig 1) [9,10]. Two major families of canonical torso structures have been identified, and are referred to as “bulged” and “non-bulged” torsos [10]. In this study, the geometries of the bulged torso domain have been used to develop restraints that restrict the sampling space of the HCDR3 torso and result in more native-like models when *de novo* modeling the entire HCDR3 loop.

Previous studies have used restraints to model the bulged HCDR3 torso, following rules previously described by Shirai et al. wherein a pseudodihedral angle restraint was calculated from the Cα atoms of residues T5, T6, T7 and the following initial residue of Framework 4 to define the bulged or non-bulged torso [18,21–24]. Weitzner et al. [18] utilized RosettaAntibody implemented within the Rosetta 3 framework to predict the structures of 11 previously unpublished antibody structures for the second antibody modeling assessment (AMA-II) [21]. The longest HCDR3 loop in AMA-II contained 16 residues, and was predicted by the RosettaAntibody team with an RMSD of 3.70Å to the native HCDR3 loop [18, 21]. Shirai et al. also competed in AMA-II, and used their torso restraint rules to filter results generated by a pipeline that includes both Spanner and OSCAR for loop structure prediction; in comparison to the RosettaAntibody team described above, their best model for the longest HCDR3 loop had an RMSD of 3.29Å to the native HCDR3 loop [21, 23].

In this study, a novel set of restraints was tested on 28 previously crystallized human antibodies with HCDR3 loops of increasing length and structural complexity. We expect that these restraints will improve modeling of antibodies for which no structural information is available, providing a means by which comprehensive structural studies of antibodies may be accomplished.

## Results

### Measuring bulged and non-bulged torso dihedral angles

An annotated list of antibodies was used to cull experimentally derived structures from the Protein Data Bank (PDB), expanding upon the list published by North et al. [10]. Following the IMGT conventions for defining the HCDR3, where the first HCDR3 residue occurs immediately following the V-gene residue Cys104 and the last HCDR3 residue occurs immediately preceding the J-gene residue Trp118, the torso is defined as the first three and the last four residues of the HCDR3 [10,25]. Accordingly, torso domain regions were pulled from these structures as two short peptide fragments (T1-T3 and T4-T7) and clustered using Rosetta at a threshold of 2 Å to separate bulged and non-bulged torsos. Previous studies identified a sequence motif (Arg or Lys at T2 and Asp at T6) that contributes to bulged torso formation in some but not all cases; these key residues were conserved in our bulged cluster, with 80% of bulged structures presenting Arg or Lys at T2, 73% presenting Asp at T6, and 65% retaining the complete T2/T6 sequence motif (S1 Fig) [9,10]. We found that germline-encoded regions of the antibody sequence often contribute these critical residues, as the end of the V gene segment contributes the first two to three torso residues while the J gene segment contributes the last four torso residues. The T2/T6 sequence motif that is often found in bulged torsos is present in 73% of naïve V and 92% of J germline gene allele segments (S1 Fig).

The φ and ψ angles of the seven torso residues of each antibody structure were measured, with key differences between bulged and non-bulged torsos identified in the ψ angles of residues T4 and T6 (Table 1). However, upon further study of previously defined torso clusters we observed that the ψ angle of T4 is able to form two distinct conformations in both bulged and non-bulged torso clusters, and the T4 ψ angle does not distinguish between bulged and non-bulged torso clusters; the differences we observed when comparing all bulged antibodies to all non-bulged antibodies were due to the limited sample size of structures available in the PDB for these sub-conformations (S2 Fig) [10]. This is in contrast to for example T5, where a larger standard deviation is observed but still a statistically significant preference for a smaller ψ angle in a bulged torso exists. Average φ and ψ angles were calculated as follows:

$$atan2\left(\frac{\sum \sin\alpha }{n},\frac{\sum \cos\alpha }{n}\right)$$ (1)

An approximate standard deviation was found using the following equations. For the vector *v*:

$$\underset{v}{\to }=\left(\frac{\sin\alpha }{n},\frac{\cos\alpha }{n}\right)$$ (2)

Approximate standard deviation is calculated using:

$$\sqrt{2\times [1-\underset{v}{\to }]}$$ (3)

It is worth noting that straightforward average and standard deviation calculations are insufficient when handling circular values such as dihedral angles.

**Table 1:** Bulged and non-bulged dihedral angle measurements.

Torso Residue	Bulged		Non-bulged	
	φ	ψ	φ	ψ
T1	-145 ± 9	148 ± 12	-146 ± 12	145 ± 16
T2	-101 ± 22	142 ± 13	-109 ± 20	136 ± 26
T3	-107 ± 32	137 ± 33	-119 ± 44	138 ± 51
T4	-121 ± 49	161 ± 48	-82 ± 49	3 ± 59
T5	-95 ± 35	98 ± 26	-126 ± 43	136 ± 53
T6	-87 ± 18	-30 ± 26	-118 ± 34	129 ± 24
T7	-126 ± 14	134 ± 10	-125 ± 19	136 ± 11

The average and standard deviation of φ and ψ angles were calculated from existing human and mouse antibody crystal structures available in the PDB. Torso structures were clustered as bulged (n = 218) and non-bulged (n = 38) using a cluster radius of 2 Å.

### Derivation of restraints for bulged torso conformation

It has been observed that Rosetta rarely samples the bulged torso conformation when modeling HCDR3 loops [14]. Due to this limitation, coupled with the greater amount of experimentally derived structural data available for bulged torsos than non-bulged torsos and the fact that bulged torsos are more prevalent in the human antibody repertoire, we chose to focus on developing restraints to improve modeling of HCDR3 loops with bulged torsos. Rosetta uses a defined format to read in experimentally derived restraints. We used our measurements to generate dihedral angle restraints following a circular harmonic scoring function. Since the ψ angle measurement of T4 varies by 180 degrees between known bulged torso clusters, this measurement was omitted from our calculated restraints (S2 Fig).

### Modeling HCDR3 loops using bulged torso restraints

Following the protocol capture outlined in Supplemental Information, these restraints were used to model and score the HCDR3 loops from 28 benchmark antibodies whose structures had been previously determined by X-ray crystallography (Table 2). These 28 benchmark structures represent HCDR3 lengths from 11 to 26 residues, with a mean length of 16 residues, spanning a range regularly observed in human antibody repertoires that also have a mean HCDR3 length of 16 amino acids [26]. Each of the benchmark antibodies was crystallized in the absence of an antigen (*i*.*e*., apo) in order to avoid attempts to model conformations achieved by induced fit with a binding partner.

**Table 2:** Experimentally derived antibodies used to benchmark bulged torso restraints.

PDB ID	HCDR3 Length	Resolution (Å)	Source
1WT5	11	2.10	*Humanized*
2G75	11	2.28	*Human*
4G5Z	11	1.83	*Human*
3QRG	12	1.70	*Human*
4G6K	12	1.90	*Humanized*
4LLU	12	2.16	*Human*
1FVC	13	2.20	*Humanized*
3HI5	13	2.50	*Human*
4HFW	13	2.60	*Human*
4FQH	14	2.05	*Human*
4NM4	14	2.65	*Human*
8FAB	14	1.80	*Human*
3G6A	15	2.10	*Human*
3TNM	15	1.85	*Human*
3W9D	15	2.32	*Human*
1AQK	16	1.84	*Human*
1DQL	16	2.60	*Human*
1OM3	16	2.20	*Human*
1U6A	17	2.81	*Human*
3AAZ	17	2.20	*Humanized*
4M5Y	17	1.55	*Human*
3INU	18	2.50	*Human*
3QEH	18	2.59	*Human*
4F58	18	2.49	*Human*
1HZH	20	2.70	*Human*
4LKC	22	2.20	*Human*
1RHH	24	1.90	*Human*
4FNL	26	2.30	*Human*

28 high-resolution antibody structures solved by X-ray crystallography were used to benchmark the bulged torso restraints. Each of these antibody structures was solved in the absence of antigen (*i*.*e*., apo structures) and all residues in the HCDR3 loops were resolved.

**Fig 2:**
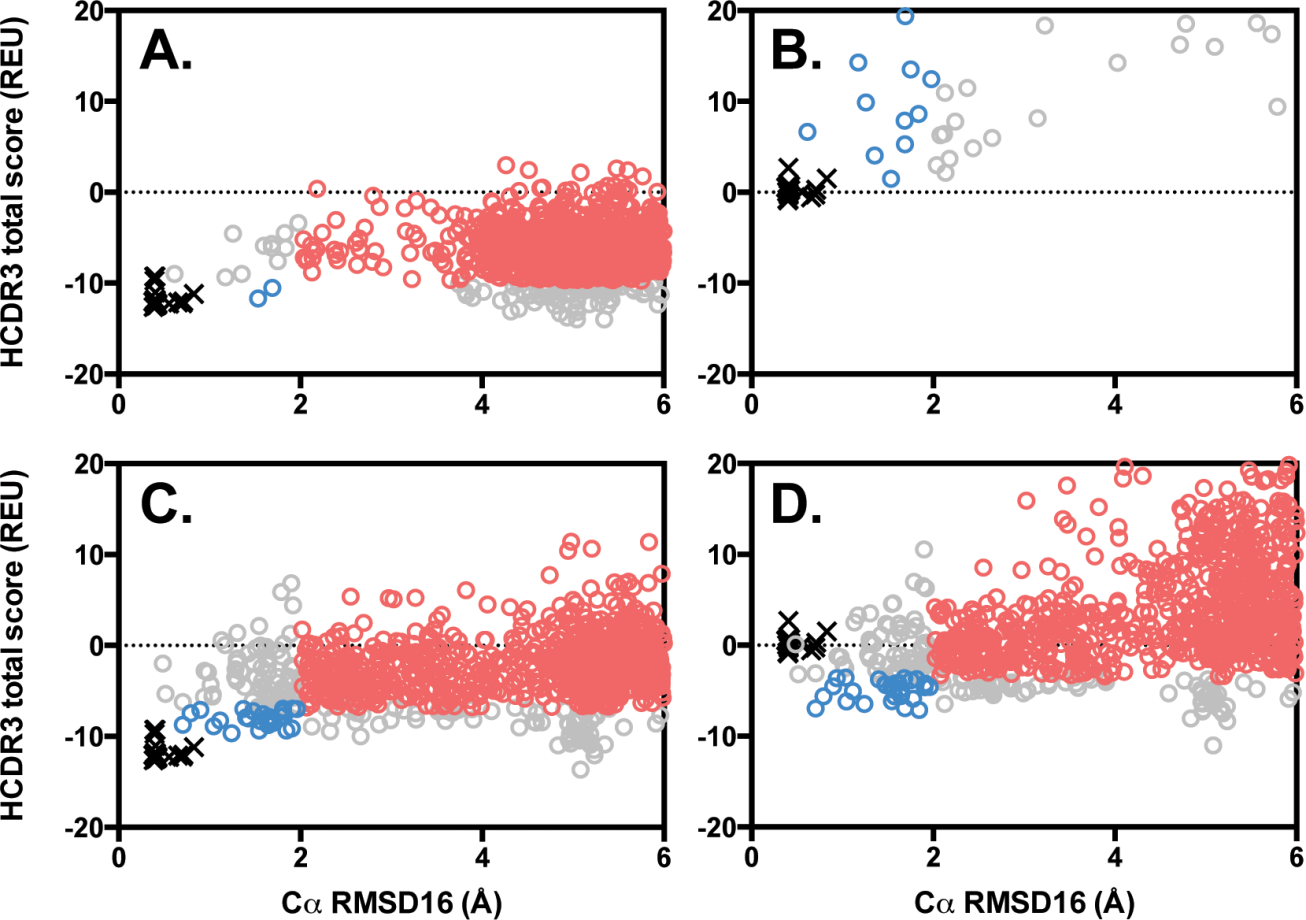
Bulged torso restraints improve native-like HCDR3 sampling and recovery. Using Rosetta LoopModel, 1,000 models of the benchmark antibody 4G5Z (circles) were generated with or without bulged restraints and these models were then scored with or without bulged restraints (panel A, modeled and scored without restraints; panel B, modeled without but scored with restraints; panel C, modeled with but scored without restraints; panel D, modeled and scored with restraints). The native crystal structure 4G5Z was also minimized using Rosetta FastRelax, generating 20 structures (black x’s). The total HCDR3 score (in Rosetta Energy Units, or REU) is shown versus the Cα root mean square deviation of the HCDR3 loop, normalized to that of a protein loop containing 16 residues (RMSD16, in Å) to the native crystal structure. Models with scores ranked in the top 10% and RMSD16 ≤ 2 Å have been colored blue, while models with scores ranked below the top 10% and RMSD16 > 2 Å have been colored red. Improved native-like HCDR3 sampling is observed as a greater density of low RMSD16 models (blue circles) in comparison to Panel A, while improved model recovery is defined as a greater correlation between RMSD16 and score (colored vs. gray circles) in comparison to Panel A, as seen in panels C and D.

Restraints function as a penalty during Rosetta’s scoring protocol, *i*.*e*., a positive energy value is added when a dihedral angle leaves the allowed range. In this case, models formed with native-like bulged torso dihedral angles would have no (or only a very small) penalty from the restraint term, whereas models that deviated from the bulged torso dihedral angles would be penalized with a positive energy score. When restraints were applied during modeling, we observed a higher density of low-scoring, low-RMSD models (Fig 2C, blue circles, n = 26) than when modeling without restraints (Fig 2A, blue circles, n = 2). These low-scoring, low-RMSD models are defined as scoring in the top 10% of models, with Cα RMSD16 to the native structure of ≤ 2 Å (represented as blue circles, whereas models scoring below the top 10% of models with Cα RMSD16 > 2 Å are represented as red circles in Fig 2 and S2 Fig. When restraints were applied during scoring but not during modeling (Fig 2B) we found that the resulting models incur substantial restraint penalties due to non-native-like sampling of the torso domain, however the correlation between score and RMSD16 is improved. During application of this protocol wherein a native structure is unavailable, the ability to identify native-like models by score alone is extremely valuable. When applying restraints during both modeling and scoring, Rosetta generates a model population where an increased number of native-like structures correlate with low scores (Fig 2D, blue circles, n = 30; also see S1 File) as compared to experiments modeled and scored without restraints (Fig 2A, blue circles, n = 2; also see S1 File). Finally, we found that the application of these restraints results in more models whose backbone structures agree with bulged torso measurements defined in the literature (n = 719 with restraints, n = 33 without restraints; see S3 Fig) [14,22].

**Fig 3:**
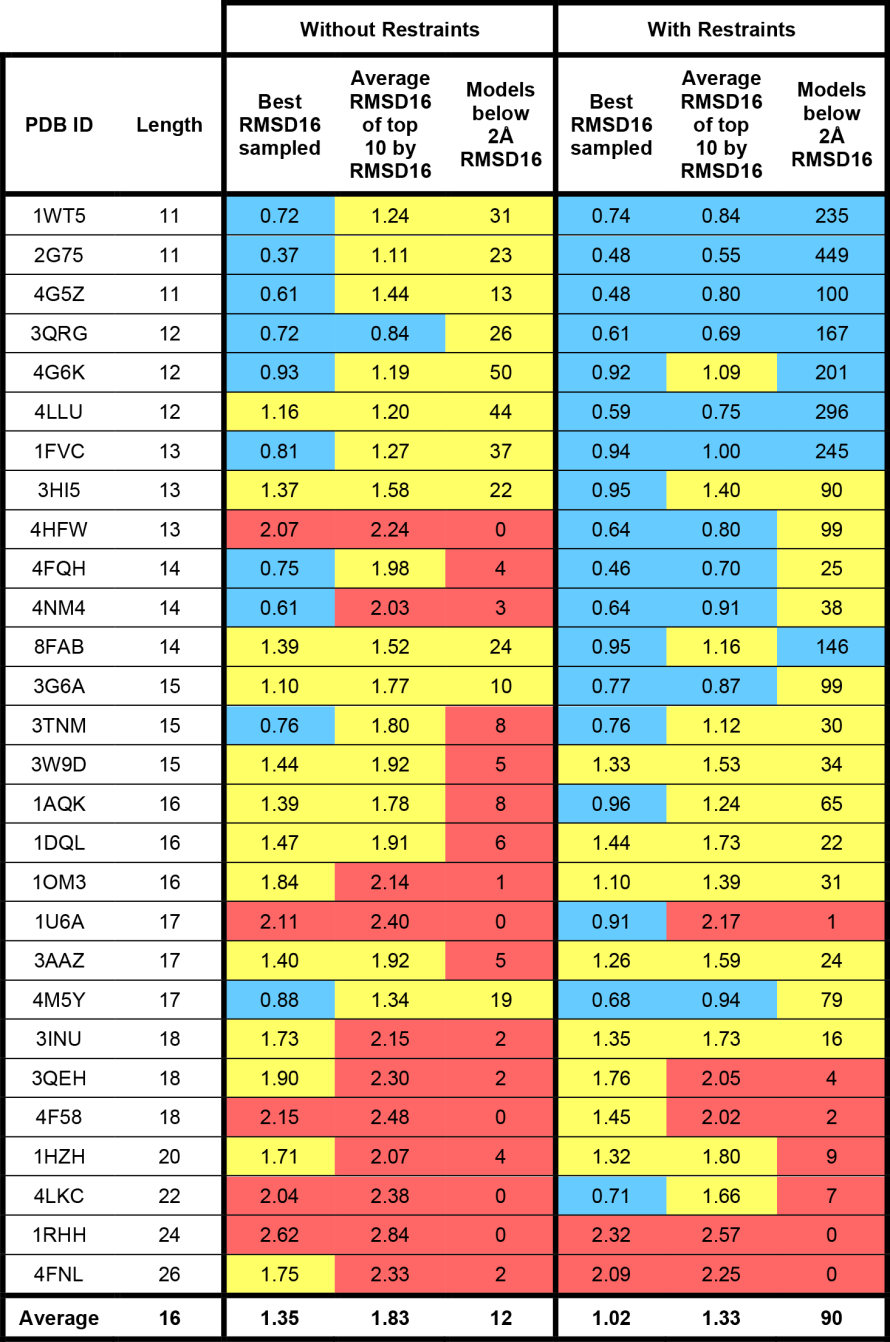
Torso restraints improve sampling of bulged HCDR3 loops. For each benchmark antibody structure, 1,000 models were generated with or without bulged torso restraints. The number of models below 2 Å RMSD16 to the native structure, the best RMSD16 sampled, and the average RMSD16 of the best 10 models ranked by RMSD16 are provided. For RMSD16-containing cells, blue shading represents RMSD16 ≤ 1 Å; yellow shading represents RMSD16 between 1 and 2 Å; red represents RMSD16 > 2 Å. For cells containing the number of models below 2 Å, blue shading represents ≥ 100 models; yellow shading represents ≥ 10 models; red shading represents fewer than 10 models.

**Fig 4:**
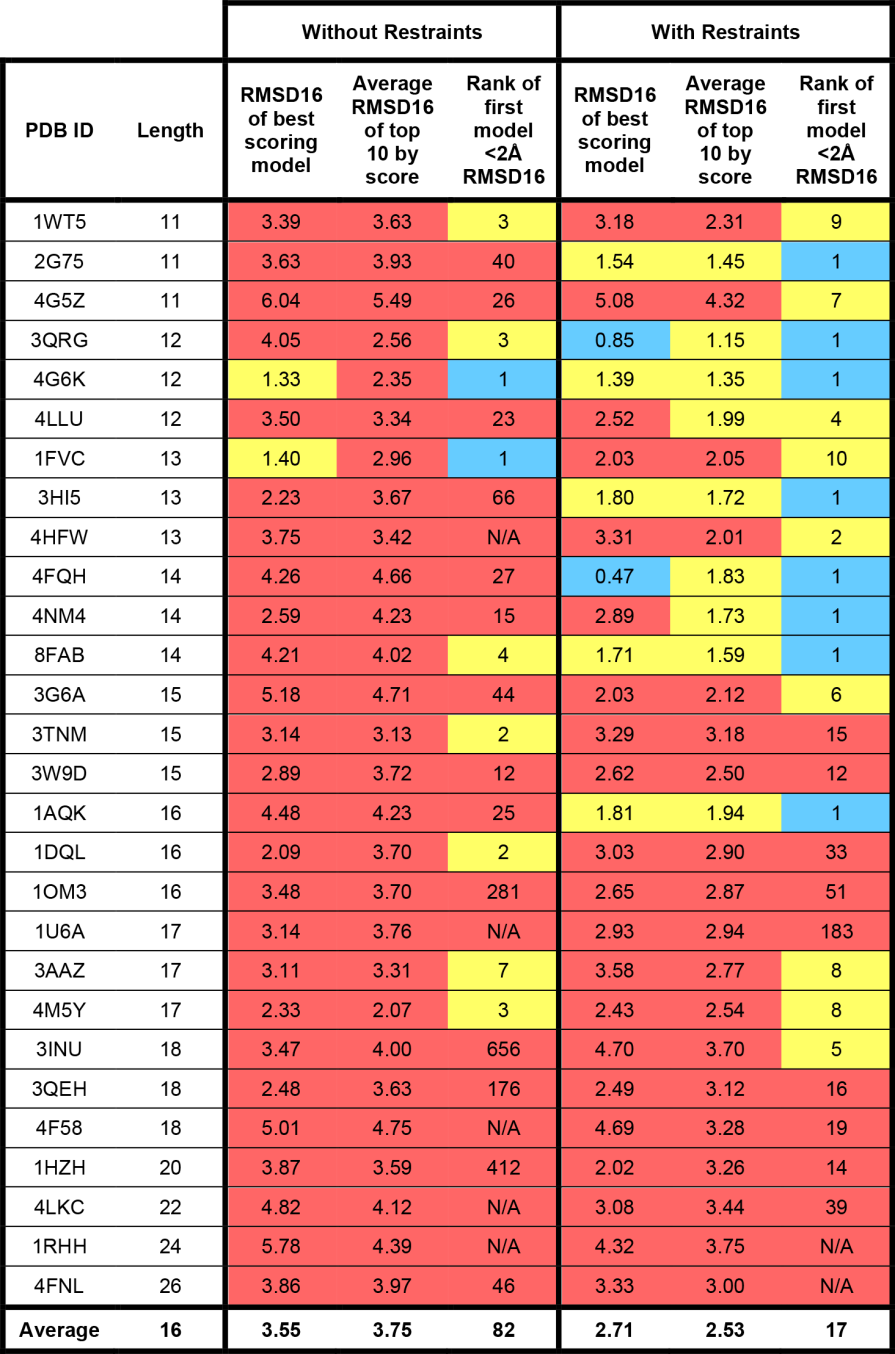
Torso restraints improve recovery of native-like bulged HCDR3 loops. For each benchmark antibody structure, 1,000 models were generated with or without bulged torso restraints. The number of models below 2 Å RMSD16 to the native structure, best RMSD16 sampled, average RMSD16 of the best 10 models ranked by RMSD16, RMSD16 of the best model ranked by Rosetta score, average RMSD16 of the top 10 models ranked by Rosetta score, and the rank of the first model below 2 Å when sorted by Rosetta score are provided. For RMSD16-containing cells, blue shading represents RMSD16 ≤ 1 Å; yellow shading represents RMSD16 between 1 and 2 Å; red represents RMSD16 > 2 Å. For rank-containing cells, blue shading represents rank 1; yellow shading represents ranks 2 to 10; red shading represents ranks > 10.

**Fig 5:**
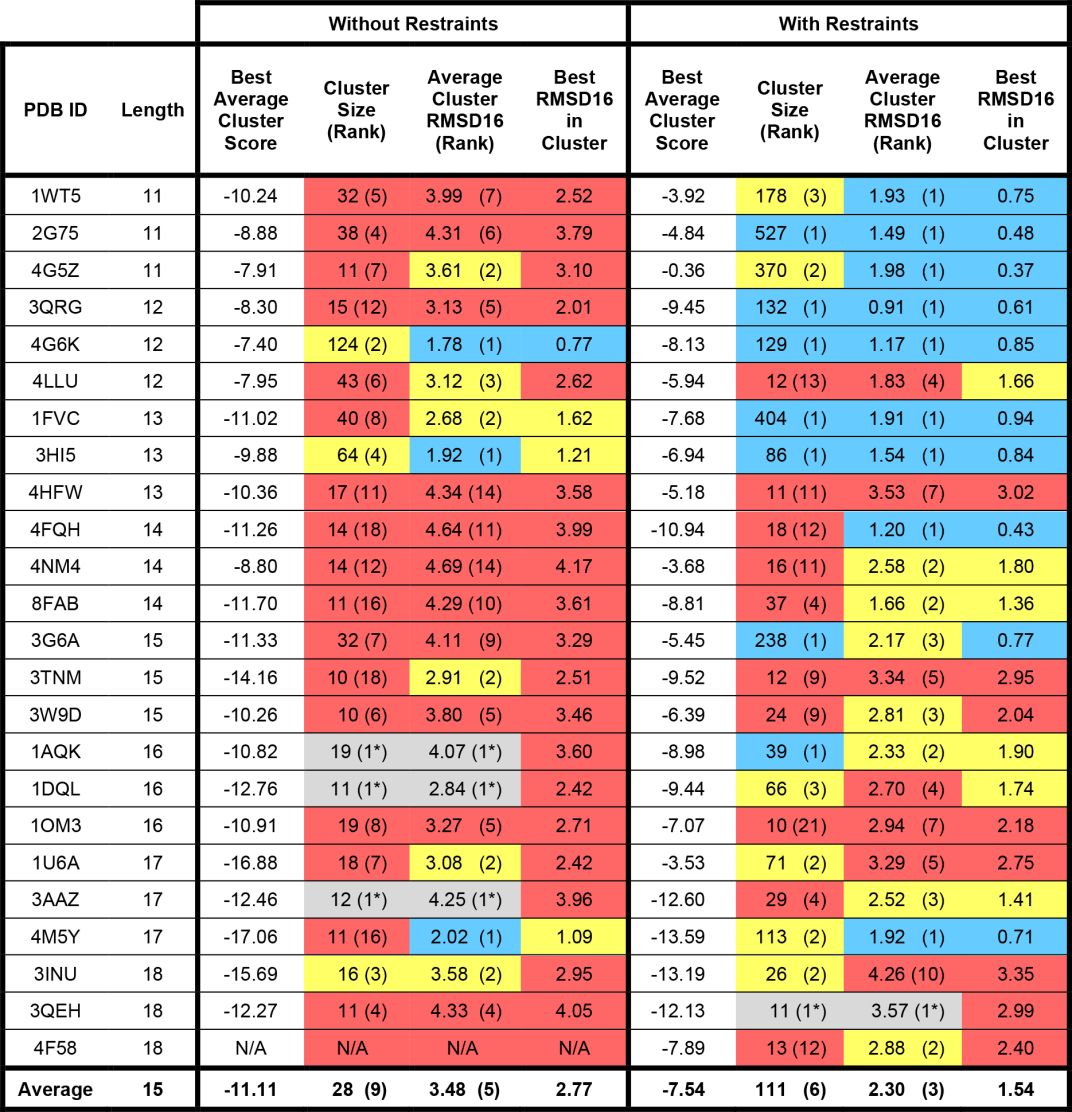
Cluster analysis of bulged HCDR3 loop modeling. Calibur was used to cluster the 1,000 models generated with or without bulged torso restraints for each antibody, using a threshold of 2.0. Clusters containing less than 1% of the total models were omitted from analysis; models generated for benchmark antibodies 4F58, 1HZH, 4LKC, 1RHH and 4FNL did not produce any large clusters upon analysis (N/A). Average Rosetta score was calculated for each cluster, and the cluster with the lowest average score was selected as the “correct” cluster. The size of this correct cluster (and it’s rank among cluster sizes), its average RMSD16 to the native structure (and rank among average RMSD16 measurements) are provided. Cells containing rank data are shaded blue if the value represents the top rank, yellow for ranks 2–3, and red for ranks >3; if only one cluster (1*) was found, the cell is shaded gray. For RMSD16-containing cells, blue shading represents RMSD16 ≤ 1 Å; yellow shading represents RMSD16 between 1 and 2 Å; red represents RMSD16 > 2 Å. Values were omitted from column averages if ≤1 cluster was found.

The results of modeling the 28 benchmark HCDR3 loops with or without bulged torso restraints can be found in Figs 3–5. We observed changes in both conformational sampling and in model discretion by score when restraints were applied. To analyze improvements in conformational sampling, models were ranked by RMSD16 to their native structure (Fig 3) and to study changes in scoring discretion the models were ranked by HCDR3 score (Fig 4). Finally, models were clustered using a package called Calibur and the best cluster by average HCDR3 score was analyzed (Fig 5).

### Bulged HCDR3 restraints improve native-like conformational sampling

Modeling with bulged torso restraints improved native-like conformational sampling (the number of models with RMSD16 below 2 Å) in 26 out of 28 benchmark cases (Fig 3); in the remaining case of benchmark antibody 1RHH with an HCDR3 loop of 24 residues, no models below 2 Å were observed when modeling with or without restraints, and in the case of 4FNL with an HCDR3 loop of 26 residues, 2 models below 2 Å were observed when modeling without restraints, compared to no models sampled below 2 A when modeling with restraints. On average, 90 models below 2 Å were generated with restraints, compared to only 12 models below 2 Å without restraints. The best RMSD sampled using bulged torso restraints was below 1 Å in 18 out of 28 cases with restraints, compared to 10 out of 28 cases without restraints. The average difference in the best RMSD sampled was 0.33 Å lower when restraints were applied during modeling. Furthermore, the average RMSD16 of the most native-like 10% of models (when ranked by RMSD16) is below 1 Å in 11 out of 28 cases when restraints are applied, compared to just 1 of 28 cases without restraints, revealing improved depth of high-resolution native-like sampling.

State-of-the-art computational methods to construct loop regions in proteins work reliably until about eight residues, and provide good results from some loops up to twelve residues [18–21]. Beyond this limit, the conformational space often becomes too large to be sampled exhaustively. Many HCDR3 loops are longer and specialized methods are needed to limit the conformational space. Our analyses describe better sampling of native-like structures during modeling of these diverse HCDR3 loops when our torso restraints are used, with qualitative changes in performance observed at 14 and 18 amino acids.

### Bulged HCDR3 restraints improve scoring discretion

The ability to identify native-like HCDR3 loops by score when *de novo* modeling using Rosetta is of critical importance. Unfortunately, we found the predictive ability of Rosetta’s scoring function in the absence of restraints to be lacking; when ranking models by HCDR3 score, only 2 of 28 benchmark cases resulted in a top-scoring model with RMSD16 < 2 Å (Fig 4). However when restraints were applied, ranking models by score resulted in 7 of 28 cases with an RMSD16 below 2 Å and two of those with RMSD16 below 1 Å (antibody 3QRG, 12 amino acids long and 4FQH, 14 amino acids long). On average, the RMSD16 of the best scoring model improved by 0.84 Å when restraints were used during modeling and scoring. Because restraints improve sampling, there was also a marked improvement in the average RMSD16 of the top 10 models ranked by score; when restraints are applied, the average is below 2 Å in 9 out of 28 cases, but no results below 2 Å were found when restraints were not used. On average, there is an improvement of 1.22 Å in the average RMSD16 of the top 10 models ranked by score. The average rank of the first model below 2 Å is 17 when restraints are applied and in 8 of 28 cases the first-ranking model is below 2 Å, compared to only 2 out of 28 cases resulting in a first-ranking model below 2 Å and an average rank of 82 when restraints are not used. Altogether these analyses reveal that the bulged torso restraints improve scoring discretion of native-like structures, but that further improvement to the scoring of HCDR3 loops is needed [27].

### Clustering bulged HCDR3 loop models

Using the clustering package Calibur [28], we analyzed the HCDR3 models generated with and without bulged restraints (Fig 5). Only clusters containing >1% of models (10 or more) were considered. For models made based on structures with 20 or more amino acids in the HCDR3 loop, no sufficiently large clusters were found. For the other benchmark structures, clusters were sorted by average cluster HCDR3 score, with the lowest average HCDR3 score being chosen as the “correct” cluster. This approach to selecting the “correct” conformation is common when *de novo* modeling HCDR3 loops, as the native structure of the loop is not known outside of benchmark studies. When restraints were used during modeling, the rank of the cluster size (how large a cluster is compared to other clusters) improved in 18 out of 24 cases over experiments where restraints were not used. When restraints were applied during modeling, the average RMSD16 of the correct cluster improved in 21 out of 24 cases. The average RMSD16 for the best cluster by score was top-ranking in 9 out of 24 cases when restraints were applied during modeling, compared to just 3 out of 24 cases when restraints were not used, which reveals the predictive power of our scoring metrics when restraints are applied.

## Discussion

There is a growing body of work surrounding canonical structures of antibody CDR loops, first described by Chothia and colleagues and updated as recently at 2011 by the Dunbrack group [9,10]. These groups have shown that that five of the six CDR loops take on canonical structures, and that the remaining HCDR3 forms only a few canonical classes of structure in its torso domain. Our work builds upon this background, and has led to the development of knowledge-based structural restraints from available crystal structures of HCDR3 loops with bulged torsos. We have shown that these restraints can be used to restrict the sampling space Rosetta searches during *de novo* loop modeling, limiting the torso domain to the φ and ψ angles of these residues that have been experimentally observed. These torso restraints improve native-like structure sampling and score-based differentiation of native-like HCDR3 models. We have also shown that such structural restraints improve Rosetta’s ability to model longer HCDR3 loops than previously possible, extending the range of the technique to cover more biologically relevant HCDR3 loop lengths.

While this study focuses on benchmarking new knowledge-based restraints against antibodies whose structures have been experimentally determined, the true value of these restraints is in their ability to improve *de novo* antibody modeling. Such antibody structural predictions are a more rapid approach than experimental structural techniques, and can improve our understanding of host-pathogen interactions, provide insight into mechanisms of viral infection, and may lead to new monoclonal antibody therapeutics or vaccine candidates. Combined with our prior understanding of canonical CDR loops, which had made it possible to homology model much of the functional surface of the antibody (the “paratope”) using Rosetta, we can now predict the remaining HCDR3 which is critical in many antibody-antigen interactions. The central dogma of structural biology, that structure dictates function, lets us expect that improved accuracy in modeling HCDR3 will lead to improved accuracy in modeling antibody/antigen interactions which in turn leads to improved prediction of antibody function. We recognize that further experiments would be needed to prove this. Finally, upcoming advances in antibody sequencing, including the ability to sequence endogenously paired heavy and light chains, will provide the last critical insight in antibody modeling; we must now come to understand restrictions at the heavy and light chain interface that alter the paratope, and incorporate such restrictions into our structural predictions.

Although we have applied this approach to improving human antibody modeling, we recognize that this approach to structural restraint development is applicable to many other protein families in which structurally diverse surface loops with key functional importance are supported upon more structurally restricted framework regions [27]. Obvious examples include proteins with the PDZ domain and peptidase C1 domain protein families, which were found to use bulged HCDR3-like loops to recognize and bind their substrates [14]. Finally, we have shown that knowledge-based structural restraints can be calculated easily and applied to improve modeling of novel loops not previously solved by experimental techniques, provided enough experimentally derived structural data is available for framework regions of functional loops in other protein families, and that canonical classes of those regions can be defined.

## Materials and Methods

### Calculating bulged and non-bulged torso dihedral angles

A collection of antibody heavy chain variable domains was manually curated from the PDB, building upon a published list [10] (S2 File). The torso residues of these structures were extracted from the PDB files and were clustered using Rosetta Cluster with a cluster radius of 2 Å to separate bulged and non-bulged antibody torsos. φ and ψ dihedral angles of the seven torso residues were found using Biopython [29], with average and approximate standard deviation calculated using Eqs 1 and 2 (Table 1).

### Generating HCDR3 loop models

The complete protocol for generating the HCDR3 loop models using Rosetta is described in S3 File and example file input and output is provided in S4 File. In brief, structure files for each benchmark antibody were downloaded from the PDB and were cleaned such that only a single variable domain remained. Input files for loop modeling were generated with the assistance of a suite of python scripts, and fragments were selected using the fragment picker. Centroid loop modeling was accomplished using cyclic coordinate descent (CCD), followed by a kinematic closure (KIC) full-atom refinement [30–32].

### HCDR3 torso sequence analysis

Sequences of the seven torso residues were taken from each of the PDB files of the bulged antibody torso cluster found above and used to generate a WebLogo using the default webserver settings [33] (S1 Fig). A second WebLogo was generated using the sequences of the torso residues taken from the IMGT human V_H_ and J_H_ gene segments [34] (S1 Fig).

## Supporting Information

S1 Fig Bulged torso structures share similar sequences, which are germline-encoded. Previous studies identified a sequence motif in bulged torso structures, which are formed primarily via a side-chain interaction between either Arg or Lys (R/K) at T2 and Asp (D) at T6. A consensus sequence from bulged torsos culled from the PDB shows the prevalence of these residues at these positions (panel A). These residues are germline-encoded, as observed in a consensus sequence of the V_H_ and J_H_ gene segments that contribute to the torso domain (panel B).
(TIF)

S2 Fig Average φ and ψ angles observed for each torso residue in known bulged and non-bulged clusters. North et al. [10] defined seven canonical torso conformations from experimentally-determined antibody structures. Two of these clusters are considered bulged (H3-anchor-1 and H3-anchor-3; blue) and two are considered non-bulged (H3-anchor-2 and H3-anchor-5; red). φ and ψ angles are well defined for both bulged and non-bulged HCDR3 torso residues. Bulged and non-bulged torsos are differentiated by their ψ angle at T6. The ψ angle at T4 is bimodal for both bulged and non-bulged HCDR3 torsos, with ~180 degrees separating the two clusters within each definition.
(TIF)

S3 Fig Bulged torso restraints improve sampling of HCDR3 torso angles. Using Rosetta LoopModel, 1,000 models of the benchmark antibody 4G5Z were generated without (red) or with (blue) bulged restraints. The τ_101_ angle and α_101_ dihedral angle defined by Weitzner et al. [14] were calculated for each model. Gray regions of the plot denote ± 3σ of the mean angles calculated for bulged HCDR3 torsos by Weitzner et al. [14]. Improved recovery of bulged torsos was observed as a greater density of points in the center gray region when restraints were applied (n = 719), versus when no restraints were applied (n = 33).
(TIF)

S1 File Bulged torso restraints improve native-like HCDR3 sampling and recovery. As in Fig 2, 1,000 models of each benchmark antibody were generated and scored with or without bulged restraints using Rosetta LoopModel (comparable to Fig 2A and 2D). Models with scores ranked in the top 10% and RMSD16 ≤ 2 Å have been colored blue, while models with scores ranked below the top 10% and RMSD16 > 2 Å have been colored red. The native crystal structure was also minimized using Rosetta FastRelax, generating 20 structures (black x’s). The total HCDR3 score vs. the HCDR3 Cα RMSD16 to the native crystal structure is shown.
(PDF)

S2 File HCDR3 definitions file. This file contains two comma separated value tables. The first table represents the non-bulged antibody structures used to calculate dihedral angle values, and lists the PDB file, chain ID, HCDR3 start residue and HCDR3 end residue when each chain in the PDB file has been renumbered sequentially starting from 1. The second file lists these values for the bulged antibody structures used to calculate the dihedral angle values.
(TXT)

S3 File Rosetta protocol. A complete protocol has been provided, including Rosetta version number, for individuals who wish to utilize our methodology.
(PDF)

S4 File Rosetta protocol capture. This archive contains example input and output files needed to run the Rosetta protocol described in S3 File.
(ZIP)
