# Supplementary figures and images for: Improving Loop Modeling of the Antibody Complementarity-Determining Region 3 Using Knowledge-Based Restraints
Source: PLoS One. 2016 May 16;11(5):e0154811. doi: 10.1371/journal.pone.0154811 (PMC4868311; doi:10.1371/journal.pone.0154811)

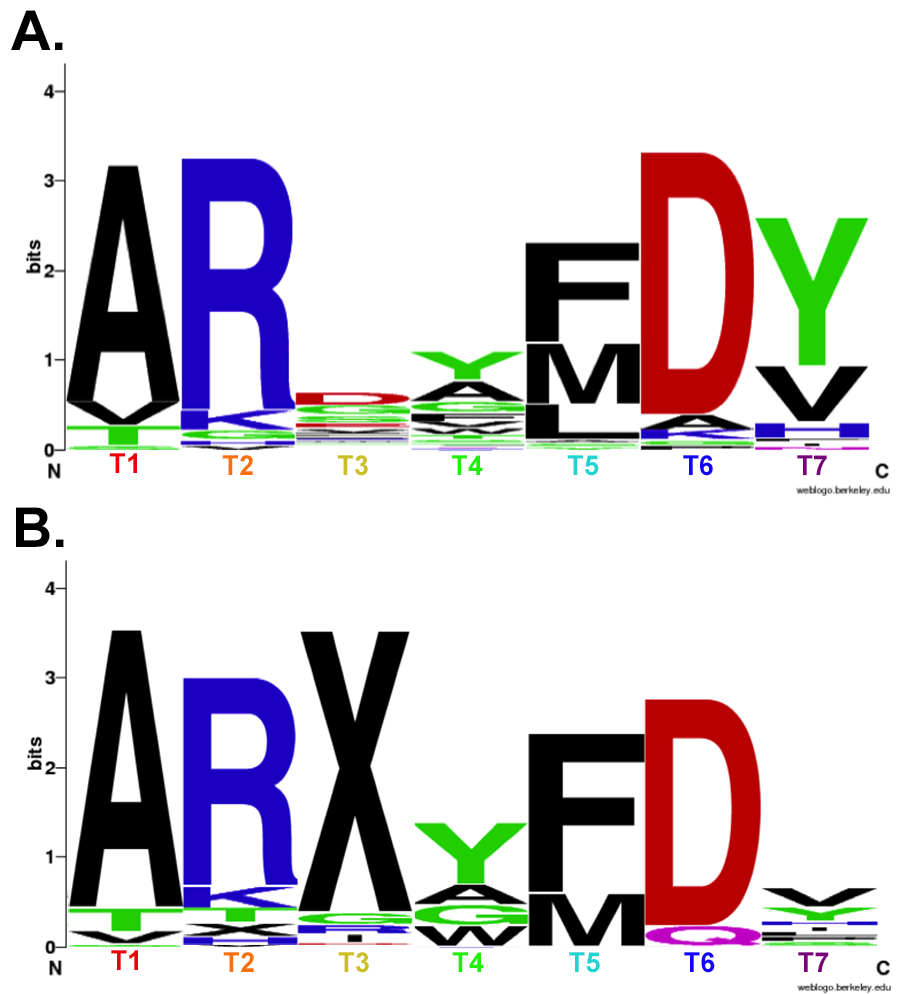

Supplement: S1 Fig — Previous studies identified a sequence motif in bulged torso structures, which are formed primarily via a side-chain interaction between either Arg or Lys (R/K) at T2 and Asp (D) at T6. A consensus sequence from bulged torsos culled from the PDB shows the prevalence of these residues at these positions (panel A). These residues are germline-encoded, as observed in a consensus sequence of the VH and JH gene segments that contribute to the torso domain (panel B). (TIF) [file pone.0154811.s001.tif]

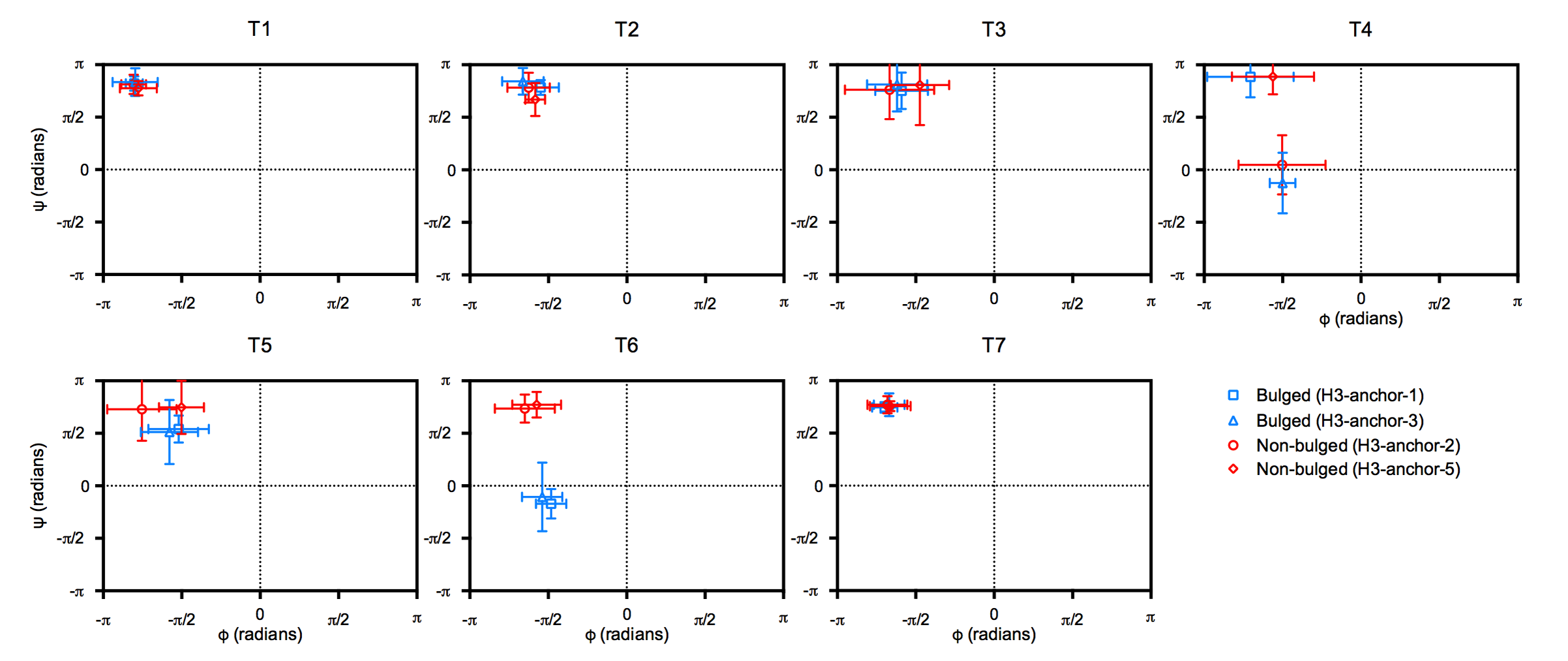

Supplement: S2 Fig — North et al. [10] defined seven canonical torso conformations from experimentally-determined antibody structures. Two of these clusters are considered bulged (H3-anchor-1 and H3-anchor-3; blue) and two are considered non-bulged (H3-anchor-2 and H3-anchor-5; red). φ and ψ angles are well defined for both bulged and non-bulged HCDR3 torso residues. Bulged and non-bulged torsos are differentiated by their ψ angle at T6. The ψ angle at T4 is bimodal for both bulged and non-bulged HCDR3 torsos, with ~180 degrees separating the two clusters within each definition. (TIF) [file pone.0154811.s002.tif]

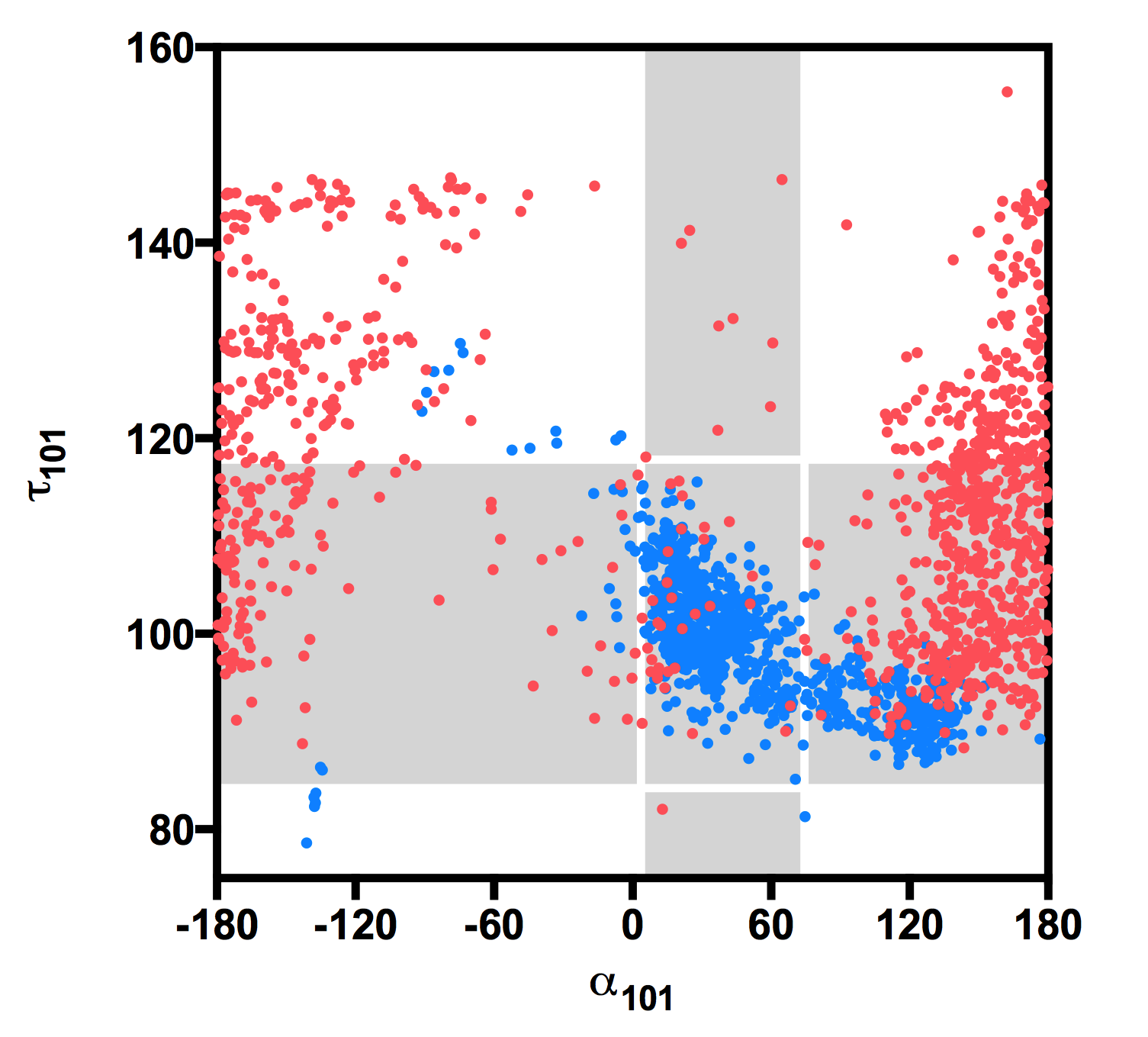

Supplement: S3 Fig — Using Rosetta LoopModel, 1,000 models of the benchmark antibody 4G5Z were generated without (red) or with (blue) bulged restraints. The τ101 angle and α101 dihedral angle defined by Weitzner et al. [14] were calculated for each model. Gray regions of the plot denote ± 3σ of the mean angles calculated for bulged HCDR3 torsos by Weitzner et al. [14]. Improved recovery of bulged torsos was observed as a greater density of points in the center gray region when restraints were applied (n = 719), versus when no restraints were applied (n = 33). (TIF) [file pone.0154811.s003.tif]

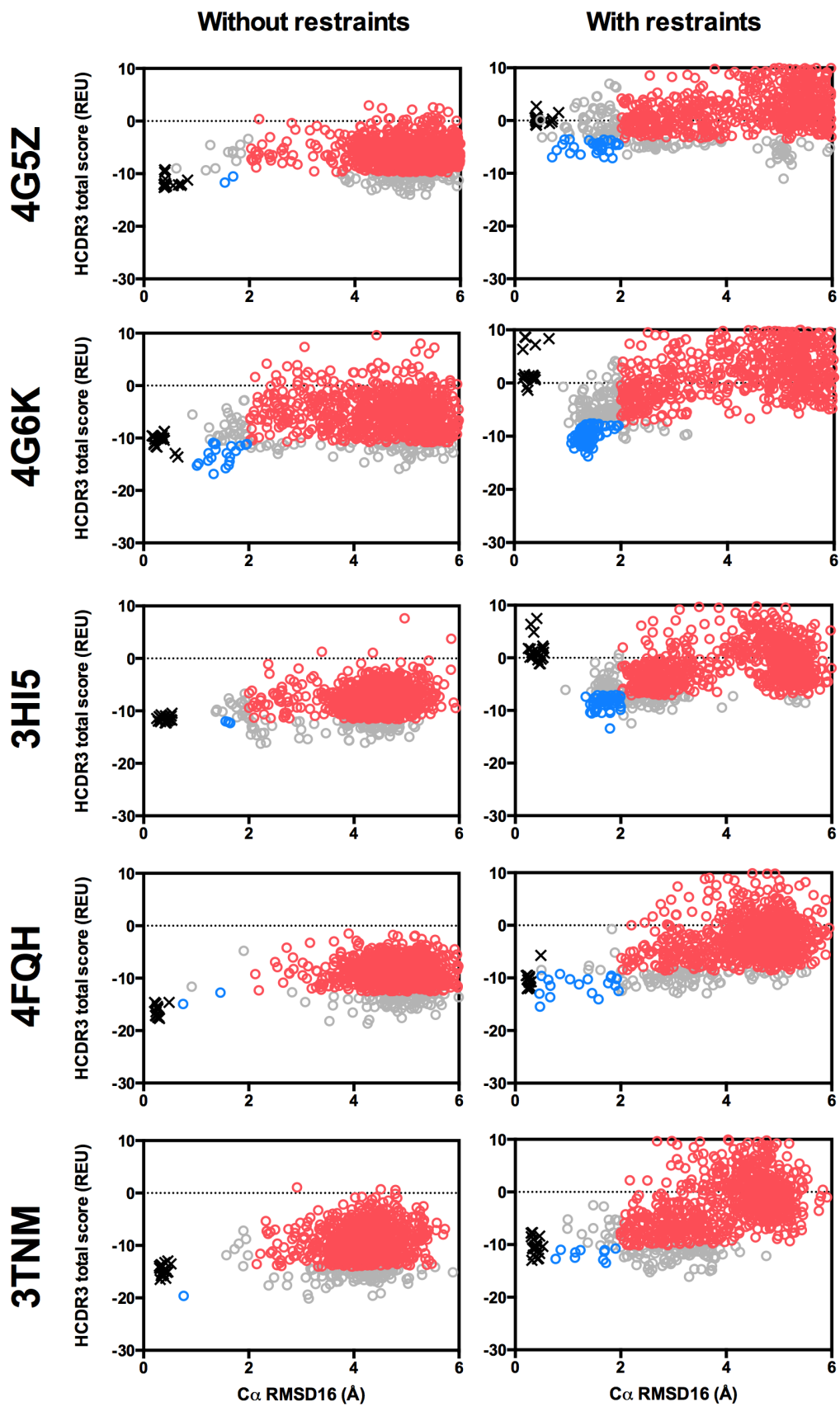

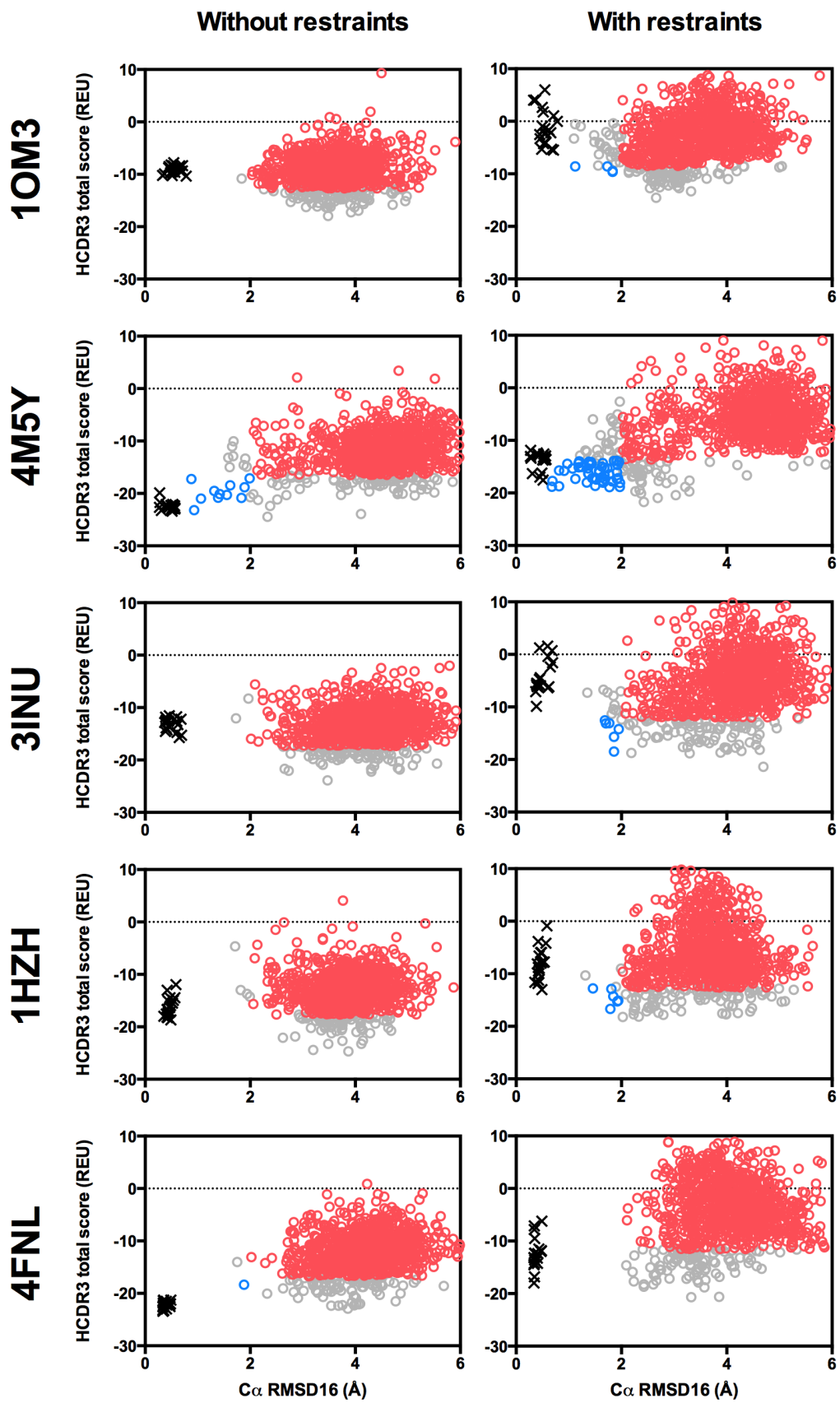

Supplement: S1 File — As in Fig 2, 1,000 models of each benchmark antibody were generated and scored with or without bulged restraints using Rosetta LoopModel (comparable to Fig 2A and 2D). Models with scores ranked in the top 10% and RMSD16 ≤ 2 Å have been colored blue, while models with scores ranked below the top 10% and RMSD16 > 2 Å have been colored red. The native crystal structure was also minimized using Rosetta FastRelax, generating 20 structures (black x’s). The total HCDR3 score vs. the HCDR3 Cα RMSD16 to the native crystal structure is shown. (PDF) [file pone.0154811.s004.pdf]
