# Supplementary material for: Improving Loop Modeling of the Antibody Complementarity-Determining Region 3 Using Knowledge-Based Restraints
Source: PLoS One. 2016 May 16;11(5):e0154811. doi: 10.1371/journal.pone.0154811 (PMC4868311; doi:10.1371/journal.pone.0154811)
Supplement: S3 File — A complete protocol has been provided, including Rosetta version number, for individuals who wish to utilize our methodology. (PDF) [file pone.0154811.s006.pdf]

Appendix I - Rosetta Protocol Capture

All Rosetta protocols were performed using version 6d19a9e478a3fc1cf369591953624a66990855ae (2013-11-15 14:37:19).

I *De novo* modeling of bulged HCDR3 loops without restraints

I.1 Prepare a PDB input file. Typically, this is accomplished by removing unnecessary chains, waters and non-protein molecules (e.g. gold) leaving behind one asymmetric unit containing a heavy and light chain. Cleaned PDB files are then renumbered using the renumber_pdb.py script:

```
/path/to/Rosetta/tools/protein_tools/scripts/pdb_renumber.py --norestart
XXXX.pdb XXXX_renum.pdb
```

I.2 Generate a FASTA sequence file from the PDB file.

```
/path/to/Rosetta/tools/protein_tools/scripts/get_fasta_from_pdb.py
XXXX_renum.pdb H > XXXX_.fasta
```

I.3 Generate a loops file (XXXX_.loops) containing one line as follows:

LOOP [residue before HCDR3 loop begins] [residue after HCDR3 loop ends] 0 0 0

```
LOOP 96 108 0 0 0
```

I.4 Generate fragments.

There are two ways to prepare files for the fragment picker: either by using the make_fragments.pl script, or by running the Robetta webserver (http://www.robetta.org/). For ease of use, the instructions below describe preparing these files using Robetta.

Submit a job to the Robetta Fragment Server by clicking the “Submit” link under Fragment Libraries from the main Robetta page (http://www.robetta.org/). Enter your registered username, the target name, and the FASTA file in the provided fields. For benchmarking purposes, select “Exclude Homologues”. Click “Submit” to place the job in queue. Jobs typically complete in less than 1 hour once they become active.

Once complete, download the following files from the webserver for use by the fragment picker:

```
XXXX_.checkpoint, XXXX_.psipred_ss2, XXXX_jufo_ss, XXXX_.homolog_vall
```

Finally, update the fragment_picker_quota.options file to point to the correct input files, then run:

```
/path/to/Rosetta/main/source/bin/fragment_picker.default.linuxgccrelease
@fragment_picker_quota.options
```

I.5 Prepare the model_wo_rest.options file to point to the correct input files, then run Rosetta LoopModel:

```
/path/to/Rosetta/main/source/bin/loopmodel.default.linuxgccrelease
@model_wo_rest.options -out:prefix ex1- >& OUT1.log &
```

II *De novo* modeling of bulged HCDR3 loops with restraints

II.1 As before, prepare a PDB input file, a FASTA sequence file, a loops file and generate fragments (Protocol I steps 1-4).

II.2 Prepare a restraint file. A script has been provided to make restraint file formatting easy, however these files can be manually created from the values available in Table 1.

```
/HCDR3_prot_capture/scripts/maketorsoconstraints.py –b –s 97 –e 107 >
XXXX_bulged.constraints
```

II.3 Prepare the model_w_rest.options file to point to the correct input files. Note the added flags for restraint file handling.

II.4 Finally, run Rosetta LoopModel:

```
/path/to/Rosetta/main/source/bin/loopmodel.default.linuxgccrelease
@model_w_rest.options -out:prefix ex2- >& OUT2.log &
```

III Comparing models generated with and without restraints

III.1 For comparison purposes, re-score the models generated in Protocol 1 using the restraint penalties. Prepare the scoring_wrest.options file to point to the correct input files, then score the models with restraints:

```
/path/to/Rosetta/main/source/bin/score_jd2.default.linuxgccrelease
@scoring_wrest.options -s ex1-*.pdb -out:prefix wrest-
```
